# Dietary recommendations for patients with chronic liver diseases: the need for increased awareness among non-hepatologist physicians

**DOI:** 10.1186/s12876-026-04685-w

**Published:** 2026-03-13

**Authors:** Yasser Fouad, Alaa M. Mostafa, Safaa M. Abdelhalim, Alshymaa A. Hassanine, Enas Kamal, Ebada Mohamed Said, Doaa Elwazzan, Nadia Abdelaaty Abdelkader, Mohammed Eslam

**Affiliations:** 1 Gastroenterology, Hepatology, and Endemic Medicine Department, Minia University, Main Road, Minia, 11432 Egypt; 2 Benha University, Hepatology & Gastroenterology, Benha, Egypt; 3 Alexandria University, Tropical Medicine, Alexandria, Egypt; 4 Ain Shams University, Tropical Medicine, Cairo, Egypt; 5 Storr Liver Centre, Westmead Institute for Medical Research, Westmead Hospital and University of Sydney, Sydney, NSW Australia

**Keywords:** Chronic Liver Disease, Physician Awareness, Nutritional Recommendations

## Abstract

**Background& aim:**

Nutritional awareness is a significant challenge for patients with chronic liver diseases (CLD), especially for those with conditions such as ascites, hepatic encephalopathy, and fatty liver disease. This survey aims to assess nutritional awareness regarding liver disease among healthcare providers (hepatologists and non-hepatologists).

**Methods:**

A structured online questionnaire was created based on clinical knowledge and established guidelines related to nutrition in liver disease. The survey consisted of 13 questions addressing nutritional considerations for liver disease patients.

**Results:**

A total of 124 out of 362 survey recipients responded. Among them, 60.5% were hepatologists, while the remaining respondents included general practitioners, endocrinologists, and cardiologists. Hepatologists were more likely than non-hepatologists to recognize the significance of nutritional guidance for their patients, with 74.7% of hepatologists supporting this view compared to only 40.8% of non-hepatologists (*p* < 0.001). For patients with compensated cirrhosis, 82.7% of hepatologists preferred no dietary restrictions, whereas only 46.9% of non-hepatologists agreed (*p* < 0.001). Both groups favored protein restriction in cases of hepatic encephalopathy, (*p* = 0.2). Regarding patients with ascites, both hepatologists and non-hepatologists recommended salt restriction, with hepatologists showing a stronger inclination. When it comes to alcohol consumption, 80% of hepatologists and 61.2% of non-hepatologists favored restricting alcohol intake in patients with chronic liver disease (*p* = 0.03).

**Conclusion:**

There are notable differences in nutritional priorities and recommendations between hepatologists and non-hepatologists. There is a pressing need to raise awareness among non-hepatologist physicians concerning dietary recommendations for patients with chronic liver disease.

**Supplementary Information:**

The online version contains supplementary material available at 10.1186/s12876-026-04685-w.

## Introduction

Chronic liver disorders (CLD) are an increasing global health concern, impacting millions of people with ailments such as metabolic dysfunction -associated fatty liver disease (MAFLD) and chronic hepatitis B and C viruses [1, 2]. As chronic liver disease progresses, patients encounter complicated clinical and nutritional issues. Malnutrition, protein-energy wasting, and micronutrient deficiencies are common in those patients associated with poor clinical outcomes, greater hospitalization rates, and deterioration in quality of life [3–6]. Nutrition is more than just a support demand; it is a foundational inquiry for better CLD care.

Nutritional assessment is essential, especially in our community. Although Egypt is geographically part of the Mediterranean region, unlike the Mediterranean diet, Egyptian food frequently includes highly refined carbs, saturated fats, and sweets, and is low in fiber and essential nutrients, which contribute to both malnutrition and obesity-related problems in CLD patients [7]. Overall, in the CLD context, current nutrition guidelines emphasize adequate energy and protein intake, as well as a diet consistent with Mediterranean principles [3–6]. Empowering people with the knowledge they need to adopt healthy eating habits can drastically improve outcomes for chronic disease and enhance overall liver function.

While hepatologists lead the specialized management of chronic liver disease (CLD), many patients are seen and advised by general practitioners, endocrinologists, and cardiologists without referral or alongside hepatologists' advice. Aside from its systemic nature, CLD may present with metabolic and cardiac manifestations. Nutritional assessment is crucial for advice-givers, hepatologists, and non-hepatologists. As a result, a multidisciplinary approach is required, and raising knowledge of dietary management options among non-hepatologist physicians becomes necessary.

The main objective of this study is to evaluate the level of nutritional awareness regarding liver disease (cirrhosis, fatty liver disease, ascites, and hepatic encephalopathy) among hepatologists and non-hepatologists. By administering a structured survey in alignment with the recognized nutritional guidelines, the survey highlights the critical gaps in nutritional clinical understanding and practice. Ultimately, the findings highlight the crucial need for targeted educational efforts and the incorporation of nutrition-focused training into all medical practitioners engaged in liver disease management.

### Patients and methods

#### The survey

A structured online questionnaire was designed and developed based on clinical knowledge and guidelines related to liver disease nutrition, sent by email to a list of professional hepatologists and non-hepatologists of different specialities in different Egyptian universities and hospitals. The questionnaire comprises closed-ended questions, ensuring reliability by minimizing subjective interpretation and more straightforward analysis. However, two questions allowed participants to select more than one answer, likely to deem all essential nutrients for patients with ascites, where multiple factors without exception or preference.

Dichotomous responses, such as “Yes, I recommend” or “I do not recommend,” were used to assess physician recommendations and beliefs about how nutritional advice is essential in patients with liver disease. However, categorical choices, such as “No restriction” or “Partial restriction,” were used to assess dietary needs according to liver disease. Multiple choices were used for nutritional elements of dietary advice.

### Participant recruitment

Three hundred sixty-two physicians were invited to participate via email distribution lists. Inclusion criteria were broadly certified physicians involved in the care of chronic liver disease patients, including hepatologists, general practitioners, internists, endocrinologists, and cardiologists. Participation was voluntary and anonymous, and informed consent was obtained electronically.

### Statistical analysis

A pilot study was carried out before full implementation to evaluate the questionnaire's efficacy in gathering the intended data on its clarity and relevance. To refine wording and find any ambiguities or repetitions in the questions, a small group of Hepatology experts from different Egyptian institutions participated in this initial step. Furthermore, test–retest reliability was done to minimize respondent bias. Construct validity and internal consistency analyses were performed (Cronbach's alpha 0.7).

All responses were collected via Google Online Docs and exported to SPSS version 26.0 (IBM Corp.) for statistical analysis. Incomplete responses were excluded from the final analysis, resulting in a total of 124 complete responses (response rate: 34.3%).

Categorical variables were represented by using frequencies and percentages. To assess differences between hepatologists and non-hepatologists, Chi-square (χ^2^) tests and Fisher exact test (cell count < 5) were used for categorical comparisons. *P*-values < 0.05 were considered statistically significant.

## Results

### Nutritional awareness among healthcare practitioners

Among 362 physicians invited to participate via email distribution lists, 124 complete respondents were validated, 60.5% were hepatologists, and 39.5% weren't (Table 1). Most respondents (56.5%) evaluated an average of 1–20 patients with liver disease per month, while 13.7% reported assessing more than 50 patients per month. 61.3% of respondents strongly believed nutrition was important, while only 1.6% did not.

**Table 1:** Nutritional awareness among healthcare practitioners

		N 124 (%)
Specialty	HePatologist	75 (60.5)
	Non-HePatologist	49 (39.5)
Number of patients (average) with liver disease assessed/month	1–20	70 (56.5)
	21–30	16 (12.9)
	30–50	16 (12.9)
	> 50	17 (13.7)
	Could not count/refused	5 (4)
Believe nutrition advice is essential in hepatic patients	Not at all	2 (1.6)
	Somewhat believe	4 (3.2)
	Yes I believe	42 (33.8)
	Strongly believe	76 (61.3)
Believe which nutritional elements are essential in patients with liver disease Response (one/more answers)	All nutrient are important	22
	Proteins	79
	Carbohydrates	62
	Fats	32
	Micronutrients (vitamins and minerals)	94
	I am not sure	1
Nutritional advice for patients with liver disease	Not at all	2 (16)
	If patient ask/according to liver disease	11 (8.9)
	Most of the patients	31 (25)
	All of them	77 (62.1)
	I am not sure	3 (2.4)
Recommendation for fat intake in patients with liver diseases	No restriction	4 (3.2)
	According to liver disease	69 (55.6)
	Partial restriction	38 (30.6)
	Complete restriction	10 (8.1)
	I am not sure	3 (2.4)
Recommendation for protein intake in patients with liver diseases	No restriction	5 (4.3)
	According to liver disease	78 (62.9)
	Partial restriction	37 (29.8)
	Complete restriction	0
	I am not sure	4 (3.2)
Recommendation for carbohydrate intake in patients with liver diseases	No restriction	30 (24.2)
	According to liver disease	58 (46.8)
	Partial restriction	31 (25)
	Complete restriction	1 (0.8)
	I am not sure	4 (3.2)
Nutritional advice for patients with compensated cirrhosis	No restriction	79 (63.7)
	Partial restriction of fat/Cho	22 (17.7)
	Partial restriction of protein	19 (15.3)
	I am not sure	4 (3.2)
Nutritional advice in patients with hepatic encephalopathy	No restriction	3 (2.4)
	Partial restriction of fat/cho	5 (4)
	Partial restriction of protein	109(87.9)
	I am not sure	7 (5.6)
Nutritional advice in patients with ascites Response (one/more answers)	No restriction	4
	Partial restriction of fat/cho	0
	Partial restriction of protein	16
	Salt restriction	105
	Blank/No special recommendation	5
Nutritional advice in patients with fatty liver disease	No restriction	0
	Partial restriction of fat/Cho	40 (32.3)
	Mediterranean diet recommendation	81 (65.3)
	I am not sure	3 (2.4)
Recommendation of 1–3 cups of coffee/day in patients with fatty liver disease	No, I do not recommend coffee intake	14 (11.3)
	Yes, I recommend 1–3 cups/day	74 (59.7)
	I am not sure	36 (29)
Recommendation of Antioxidant in patients with fatty liver disease	No, I do not recommend antioxidant	5 (4)
	Yes, I recommend antioxidant	116 (93.5)
	I am not sure	3 (2.4)
Recommendation of alcohol restriction in alcohol intake in patients with chronic liver disease	No restriction	5 (4)
	Partial restriction	29 (23.4)
	Complete restriction	90 (72.6)

Hepatologists were more likely than non-hepatologists to believe in the relevance of nutrition guidance for their patients (74.7% vs. 40.8%, *p* < 0.001) (Table 2). 68% of hepatologists advocated nutritional advice for all patients with CLD, and 9.3% preferred tailored advice according to liver disease, with no significant difference in concept between both groups (p 0.2).

**Table 2:** Comparative results between healthcare practitioners (hepatologists and non-hepatologists) in providing nutritional advice to patients with chronic liver disease

		Hepatologist N 75	Non-hepatologist N 49	*p*-value
Believe nutrition advice is essential in hepatic patients	Not at all	0	2 (4.1)	< 0.001
	Somewhat believe	1 (1.3)	3 (6.1)	
	Yes I believe	18 (24)	24 (48.9)	
	Strongly believe	56 (74.7)	20 (40.8)	
Nutritional advice for patients with liver disease	Not at all	0	2 (4.1)	0.2
	According to liver disease	7 (9.3)	5 (10.2)	
	Most of the patients	16 (21.3)	14 (28.6)	
	All of them	51 (68)	26 (53.1)	
	I am not sure	1 (1.3)	2 (4.1)	
Recommendation for fat intake in patients with liver diseases	No restriction	4 (5.3)	0	0.003
	According to liver disease	45 (60)	24 (48.9)	
	Partial restriction	20 (26.7)	14 (28.6)	
	Complete restriction	1 (1.3)	9 (18.4)	
	I am not sure	1 (1.3)	2 (4.1)	
Recommendation for protein intake in patients with liver diseases	No restriction	3 (4)	2 (4.1)	0.7
	According to liver disease	50 (66.7)	28 (57.1)	
	Partial restriction	20 (26.7)	17 (34.7)	
	I am not sure	2 (2.6)	2 (4.1)	
Recommendation for carbohydrate intake in patients with liver diseases	No restriction	14 (18.7)	16 (32.7)	0.008
	According to liver disease	44 (58.7)	14 (28.7)	
	Partial restriction	15 (20)	16 (32.7)	
	Complete restriction	0	1 (2)	
	I am not sure	2 (2.6)	2 (4.1)	
Nutritional advice in patients with compensated cirrhosis	No restriction	62 (82.7)	23 (46.9)	< 0.001
	Partial restriction fat/Cho	9 (12)	13 (26.5)	
	Partial restriction/protein	2 (2.6)	11 (22.4)	
	I am not sure	2 (2.6)	2 (4.1)	
Nutritional advice in patients with hepatic encephalopathy	No restrictions/nutrients	3 (4)	0	0.2
	Partial restriction/fat,cho	1 (1.3)	4 (8.2)	
	Partial restriction/protein	66 (88)	43 (87.8)	
	I am not sure	5 (6.7)	2 (4.1)	
Nutritional advice in patients with fatty liver disease	Partial restriction/fat/cho	18 (24)	21 (42.9)	0.03
	Mediterranean diet	55 (73.3)	26 (53.1)	
	I am not sure	1 (1.3)	2 (4.1)	
Recommendation of 1–3 cups of coffee/day in patients with fatty liver disease	Not recommend coffee intake	4 (5.3)	10 (20.4)	< 0.001
	Recommend 1–3 cups/day	57 (76)	17 (34.7)	
	I am not sure	14 (18.7)	22 (44.9)	
Recommendation of Antioxidant in patients with fatty liver disease	No, I do not recommend antioxidant	2 (2.7)	3 (6.1)	0.4
	Yes, I recommend antioxidant	72 (96)	44 (89.8)	
	I am not sure	1 (1.3)	2 (4.1)	
Recommendation of alcohol consumption in patients with chronic liver disease	No restriction	1 (1.3)	4 (8.2)	0.03
	Partial restriction	14 (18.7)	15 (30.6)	
	Complete restriction	60 (80)	30 (61.2)	

### Nutritional advice for essential elements in patients with CLD

The most frequently recognized essential nutrients among respondents were collected by choosing more than one choice; the highest believed important nutrient was micronutrients including vitamins and minerals (94), proteins (79), carbohydrates (62), and least for fats (32); however, 22 respondents considered all nutrients to be important. 62.1% of respondents proposed standardized advice for all chronic patients. However, 8.9% chose tailored advice for specific patients (if they ask/according to liver disease) (Table 1).

**Fig. 1:**
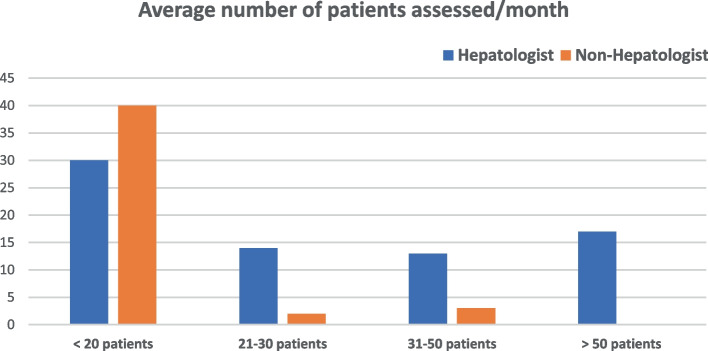
Average number of patients assessed/month by hepatologist and non-hepatologist

**Fig. 2:**
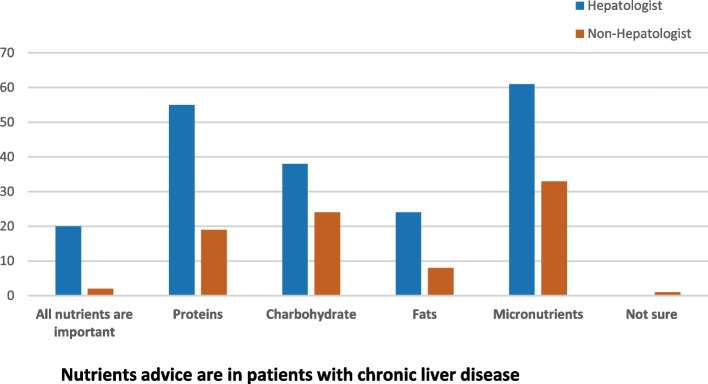
Frequency of nutritional elements essential for patients with chronic liver disease among hepatologists and non-hepatologists

Most respondents reported an evaluation average of less than 20 patients/month, while hepatologists recorded a higher average number of patients with CLD monthly (Fig. 1). Hepatologists were more likely than non-hepatologists to believe in the relevance of nutrition guidance for their patients (74.7% vs. 40.8%, *p* < 0.001), Table 2 and Fig. 2. 68% of hepatologists advocated nutritional advice for all patients with CLD, and 9.3% preferred tailored advice according to liver disease, with no significant difference in concept between both groups (p 0.2) (Fig. 2).

Regarding nutritional recommendations for fat, protein, and CHO in patients with CLD, 55.6%, 62.9%, and 46.8% of respondents recommend tailored nutritional advice according to the existing liver disease. However, carbohydrates (24.2%) were the highest recommendation for “no restriction” advice in those patients (Table 1). 60% of hepatologists stated fat consumption based on liver disease state vs. 48.9% of non-hepatologists (p 0.003).

For protein requirement, both hepatologists and non-one exhibited comparable frequencies, preferring their recommendations tailored to liver disease conditions (66.7% vs. 57.1%, p 0.7). However, carbohydrate intake, the non-hepatologists advised for “no restriction” than hepatologists (32.7% vs 18.7%), but the hepatologists preferred disease-based recommendations (58.7% vs 28.7%, p 0.008) (Table 2).

### Nutritional advice for patients with compensated and decompensated cirrhosis

In patients with compensated cirrhosis, 63.7 respondents advised their patients did not need any nutritional restriction, 17.7% and 15.3% advised partial restriction of fat/Cho and protein (Table 1). Hepatologists stated that those patients did not need dietary restrictions (82.7% vs 46.9%, *p* < 0.001). However, 26.5% and 22.4% of non-hepatologists recommend partial fat/Cho and protein restriction, respectively (Table 2).

**Fig. 3:**
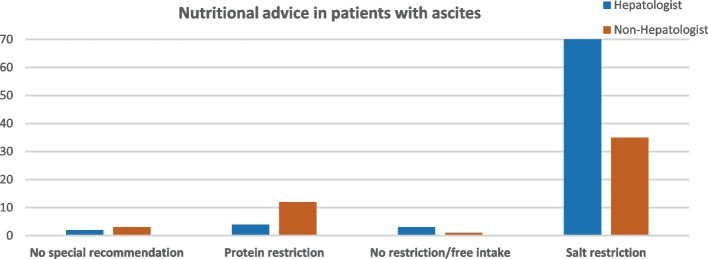
Nutritional advice provided to patients with ascites among hepatologists and non-hepatologists

Aside from nutritional recommendations for patients with decompensated cirrhosis, all healthcare practitioners supported protein restriction in patients with hepatic encephalopathy (88% vs. 87.8%, p 0.2) (Table 2). Salt restriction is the most common recommendation for patients with ascites, in particular with hepatologists supporting it more strongly (Fig. 3).

### Nutritional advice for patients with fatty liver disease

By addressing patients with fatty liver disease, 65.3% adopted the Mediterranean diet as the standard nutritional advice, 73.3% of hepatologists vs 53.1% recommended a Mediterranean diet (p 0.03) Table 2.

In addition, 76% of hepatologists endorsed 1–3 cups of coffee/day vs. 34.7% of non-one (*p* < 0.001). Antioxidants were recommended by the majority of respondents (96% hepatologists and 89.8% non-hepatologists, p 0.4). Moreover, 72.6% and 23.4% advocated comprehensive complete and partial restriction of alcohol intake in chronic liver disease, with 80% of hepatologists favoring complete restriction versus 61.2% of non-hepatologists (p 0.03).

## Discussion

Our study investigated current clinical perspectives and practices regarding nutritional awareness in chronic liver disease (CLD). While there is consensus on the significance of nutrition in improving outcomes for patients with cirrhosis and fatty liver disease, the application of this knowledge varies among healthcare practitioners. We identified a notable difference in how hepatologists and non-hepatologists implement evidence-based dietary guidelines for their patients. Hepatologists demonstrated much higher adherence to nutritional guidelines compared to their non-specialist counterparts. Additionally, they were more likely to provide dynamic, tailored dietary adjustments specific to the disease, particularly regarding protein, fat, and carbohydrate intake.

We highlighted the established nutritional needs for CLD patients with severe complications such as ascites and hepatic encephalopathy. For patients with ascites, salt restriction was noted as important, while protein restriction was recommended for those who are comatose. In the case of fatty liver disease, hepatologists typically recommend adherence to the Mediterranean diet, moderate coffee consumption, and antioxidant supplements. Moreover, they advised alcohol restriction to help slow disease progression.

Over the past fifty years, dietary habits in Egypt have changed significantly, shifting toward energy-dense, nutrient-poor foods, as discussed by Golzarand et al. [8]. These habits contrast with global nutritional recommendations that advocate for the Mediterranean diet, avoidance of processed foods, and caloric moderation, as detailed in recent guidelines published by APASL, EASL, ESPEN, and AMAGE [3–6]. Additionally, the APASL guidelines include special recommendations for protein intake (1.2–1.5 g/kg), endorsing regular meals with late-night snacks and resistance training to prevent sarcopenia [6]. The Egyptian Clinical Practice Guidelines 2022 recognized this trend and recommended a customized nutritional strategy for management [7]. Additionally, the recent African Middle East Association of Gastroenterology (AMAGE) demonstrates a region-specific framework for diagnosis and management of MAFLD with lifestyle modification as a central therapeutic pillar, reinforcing the importance of nutritional counseling in real-world clinical practice [3]. Overall, these guidelines support the necessity of appropriate nutritional therapies to improve patient outcomes, especially in cases of complications like compensated cirrhosis, ascites, hepatic encephalopathy, and fatty liver disease.

Our study found a strong belief in the importance of nutritional advice among patients with CLD, which aligns with previous research by Liu et al. [1]. This research highlighted the rising contribution of MAFLD to global liver disease morbidity, emphasizing the need for lifestyle modifications and tailored dietary recommendations, particularly in low-income countries1. A cross-sectional study by Gazineo et al. [2], also emphasized the significance of nutrition in the quality of life for patients with CLD. Furthermore, a consensus statement on nutrition in chronic liver disease from the Indian National Association for the Study stressed the need for vitamin supplementation, including vitamins A, D, E, C, and B complex [9].

According to current standards for managing compensated cirrhosis, nearly half of non-hepatologists still endorse more conservative dietary guidelines, while hepatologists prefer no dietary restrictions. The ESPEN and EASL guidelines encourage patients to consume enough calories and protein, which is crucial for most individuals with chronic liver disease, rather than avoiding specific foods. It is recommended to have three main meals and three snacks daily, with an emphasis on consuming as many fruits and vegetables as possible [4, 5].

The study also identified practical disparities in clinical settings, such as compensated cirrhosis, ascites, and hepatic encephalopathy. In cases of encephalopathy, most participants suggested restricting protein intake; both hepatologists and non-hepatologists shared this view, despite it not being entirely aligned with recent guidelines. This difference may stem from the long-standing belief that protein restriction has been a primary therapy for hepatic encephalopathy for over fifty years [10, 11]. However, recent research indicates that protein restriction does not significantly aid in the prevention or treatment of hepatic encephalopathy [12–14].

Muscles play a crucial role in buffering ammonia and supplying amino acids for gluconeogenesis, and they suffer when protein intake is restricted or inadequate. Therefore, maintaining a regular protein diet in cases of encephalopathy is important to preserve nitrogen balance and prevent muscle wasting [4, 6]. Additionally, salt restriction is standard nutritional advice and is the first-line non-pharmacological therapy for managing ascites, as recommended by the APASL guidelines on the management of ascites in liver disease [15].

In managing patients with fatty liver disease, in-line practices were observed. Most respondents recommended the Mediterranean diet, which aligns with the guidelines from APASL, EASL, ESPEN, AMAGE [3–6]. Additionally, lifestyle modifications were suggested regarding coffee consumption and the inclusion of antioxidants, both of which are supported by increasing research. The EASL guidelines identify vitamin supplements, specifically vitamin A, D, E, C, and B complex, as potentially insufficient in liver disease [3]. Moreover, coffee has been noted for its protective role, particularly in reducing the progression of fibrosis and hepatocellular carcinoma [16].

In accordance with the APASL Clinical Practice Guidelines for 2025, it's essential to provide detailed nutritional advice tailored to patients with MAFLD and those with MAFLD-related cirrhosis and hepatocellular carcinoma. Research indicates that intermittent fasting and ketogenic diets are more beneficial than prolonged fasting for these patients. In addition to the recommended Mediterranean diet for weight loss, a caloric intake of 1200–1800 kcal per day is advised, with an emphasis on avoiding added sugars (like fructose and sucrose), saturated fats, and highly processed foods [6].

### Clinical implications

This survey provides thoughtful clinical and nutritional implications in the management of patients with CLD. Despite existing international guidelines, in real-world clinics, care advisors (hepatologists and non-hepatologists) are a part of its alignment. The observed gap in nutritional knowledge underscores the need for more consistent dietary counseling across all care levels, as well as tailored advice that accounts for cultural dietary habits. Consistently incorporating structured nutritional assessment into routine evaluations of high-risk groups enables earlier identification of malnutrition, sarcopenia, and adverse dietary patterns. Overall, the survey reframes nutrition as a fundamental component of comprehensive CLD care, with direct implications for screening, decision-making, and prognostic evaluation.

This study has limitations, including a modest sample size of 124 respondents out of 362 invited and its regional scope (an Egyptian survey), which limits its generalizability to other populations. To address this, multidisciplinary care models that incorporate primary care physicians, dietitians, and hepatologists can be implemented. Furthermore, the survey did not capture more specific details, for example, a measurable alcohol quantity among respondents, addressing fluid restriction in ascitic patients, despite its frequency, as well as phenotype-based recommendations in MAFLD patients, fell outside the scope of our survey. Also, expanding the survey to include a detailed assessment of micronutrients, probiotics, and exercise would have substantially broadened its scope and introduced additional complexity beyond the intended focus. Professional programs focused on the role of diet in the development of liver disease, as well as organized medical education programs that integrate clinical nutrition and hepatology, are also recommended. Additionally, including nutritional screening and counseling in routine hepatology practice will help ensure early detection of malnutrition and dietary risks.

In conclusion, our study demonstrated a discrepancy in nutritional awareness between hepatologists and non-hepatologists. This highlights the need for targeted educational programs to improve knowledge and encourage the adoption of current dietary techniques in managing and preventing complications in patients with chronic liver disease.

## Supplementary Information

Supplementary Material 1.

## Data Availability

The datasets generated and/or analysed during the current study are not publicly available due to ethical consideration but are available from the corresponding author on reasonable request.
