# Supplementary material for: Dietary recommendations for patients with chronic liver diseases: the need for increased awareness among non-hepatologist physicians
Source: BMC Gastroenterol. 2026 Mar 13;26:211. doi: 10.1186/s12876-026-04685-w (PMC13064337; doi:10.1186/s12876-026-04685-w)
Supplement: Supplementary file 1 — Supplementary Material 1. [file 12876_2026_4685_MOESM1_ESM.pdf]

Demographics:

1. Gender: Male/ female
2. Age: (range)
3. Place of work:
    - Urban
    - Rural
4. Specialty:
    - hepatologist
    - internal medicine
    - other (cardiologist, endocrinologist, General practitioner)

Experience:

5. How many patients with liver disease do you assess / month (Average)?
    - < 20
    - 20-30
    - 30-50
    - > 50

6. How confident are you that nutrition is essential in hepatic patients?
    - Not agree at all
    - Somewhat agree
    - Yes, I agree
    - Strongly agree

7. Do you prescribe nutritional advice to patients with Liver disease?
    - Not at all
    - If the patient asked/according to liver disease
    - Most of the patients
    - All of them
    - I am not sure

8. Which of these nutritional elements are essential in patients with liver disease? (one/more answer)
    - Micronutrients (vitamins and minerals)

- Proteins
- Carbohydrates
- Fat
- All of them
- I am not sure

9. What do you recommend for fat intake in patients with liver disease:
    - No restriction
    - Variable according to liver disease
    - Partial restriction
    - Complete restriction
    - I am not sure

10. What do you recommend for protein intake in patients with liver disease?
    - No restriction
    - Variable according to liver disease
    - Partial restriction
    - Complete restriction
    - I am not sure

11. What do you recommend for carbohydrate intake in patients with liver disease?
    - No restriction
    - Variable according to liver disease
    - Partial restriction
    - Complete restriction
    - I am not sure

12. What do you recommend for patients with compensated cirrhosis?
    - No restriction
    - Partial restriction of Fats/carbohydrates
    - Partial restriction of protein
    - No special recommendation
    - I am not sure

13. What do you recommend for patients with hepatic encephalopathy?

- No restriction
- Partial restriction of Fats/carbohydrates
- Partial restriction of protein
- No special recommendation
- I am not sure

14. What do you recommend for patients with ascites? (one/more Answers)
    - No restriction
    - Partial restriction of Fats/carbohydrates
    - Partial restriction of protein
    - No special recommendation
    - Salt restriction
    - I am not sure

15. What do you recommend for patients with fatty liver disease?
    - No restriction
    - Partial restriction of Fats/carbohydrates
    - Mediterranean diet recommendation
    - No special recommendation
    - I am not sure

16. Regarding coffee intake. What do you recommend for 1-3 cups/day in patients with fatty liver disease?
    - No, I do not recommend
    - Yes, I recommend 1-3 cups/day
    - I am not sure

17. Do you recommend antioxidants in patients with fatty liver disease?
    - No, I do not recommend
    - Yes, I recommend antioxidants in patients with fatty liver disease
    - I am not sure

18. Do you recommend alcohol restriction in patients with chronic liver disease?
    - No, I don’t recommend
    - I recommend partial restrictions

- I recommend complete restriction
- Variable according to the patient
